# Expert opinion on clinical presentation, diagnosis, and treatment of infantile-onset Pompe disease: a Delphi study in Türkiye

**DOI:** 10.55730/1300-0144.6005

**Published:** 2024-11-20

**Authors:** Ekin ÖZSAYDI AKTAŞOĞLU, Aslı İNCİ, Rıdvan Murat ÖKTEM, Gürsel BİBEROĞLU, İlyas OKUR, Fatih Süheyl EZGÜ, Filiz Başak CENGİZ ERGİN, Abdurrahman AKGÜN, Nur ARSLAN, Halil İbrahim AYDIN, Ayşe Ergül BOZACI, Mahmut ÇOKER, Fatma Tuba EMİNOĞLU, Melike ERSOY, Emine GÖKSOY, Banu KADIOĞLU YILMAZ, Mehtap KAĞNICI, Mustafa KILIÇ, Neslihan ÖNENLİ MUNGAN, Burcu ÖZTÜRK HIŞMİ, Pembe SOYLU ÜSTKOYUNCU, Ayşegül TOKATLI, Ayşe Çiğdem AKTUĞLU ZEYBEK, Berrak BİLGİNER GÜRBÜZ, Sevil DORUM, Dilek GÜNEŞ, Çiğdem Seher KASAPKARA, Sebile KILAVUZ, Erdoğan SOYUÇEN, Pelin TEKE KISA, Özlem ÜNAL UZUN, Fatma Derya BULUT, Songül GÖKAY, Selen HAS ÖZHAN, Deniz KOR, Aynur KÜÇÜKÇONGAR YAVAŞ, Figen ÖZÇAY, Yılmaz YILDIZ, Harun YILDIZ, Leyla TÜMER

**Affiliations:** 1 Division of Metabolic Diseases and Nutrition, Department of Pediatrics, Faculty of Medicine, Gazi University, Ankara, Turkiye; 2 Division of Metabolic Diseases and Nutrition, Department of Pediatrics, Faculty of Medicine, Fırat University, Elazığ, Turkiye; 3 Division of Metabolic Diseases and Nutrition, Department of Pediatrics, Faculty of Medicine, Dokuz Eylül University, İzmir, Turkiye; 4 Division of Metabolic Diseases and Nutrition, Department of Pediatrics, Faculty of Medicine, Başkent University, Ankara, Turkiye; 5 Division of Metabolic Diseases and Nutrition, Department of Pediatrics, Manisa City Hospital, Manisa, Turkiye; 6 Division of Metabolic Diseases and Nutrition, Department of Pediatrics, Faculty of Medicine, Ege University, İzmir, Turkiye; 7 Division of Metabolic Diseases and Nutrition, Department of Pediatrics, Faculty of Medicine, Ankara University, Ankara, Turkiye; 8 Division of Metabolic Diseases and Nutrition, Department of Pediatrics, Bakırköy Dr. Sadi Konuk Reseach and Training Hospital, University of Health Sciences, İstanbul, Turkiye; 9 Division of Metabolic Diseases and Nutrition, Department of Pediatrics, Faculty of Medicine, Adnan Menderes University, Aydın, Turkiye; 10 Division of Metabolic Diseases and Nutrition, Department of Pediatrics, Faculty of Medicine, Selçuk University, Konya, Turkiye; 11 Division of Metabolic Diseases and Nutrition, Department of Pediatrics, Antalya Training and Research Hospital, Antalya, Turkiye; 12 Division of Metabolic Diseases and Nutrition, Department of Pediatrics, Ankara Etlik City Hospital, University of Health Sciences, Ankara, Turkiye; 13 Division of Metabolic Diseases and Nutrition, Department of Pediatrics, Faculty of Medicine, Çukurova University, Adana, Turkiye; 14 Division of Metabolic Diseases and Nutrition, Department of Pediatrics, Faculty of Medicine, Marmara University, İstanbul, Turkiye; 15 Division of Metabolic Diseases and Nutrition, Department of Pediatrics, Kayseri City Hospital, Kayseri, Turkiye; 16 Division of Metabolic Diseases and Nutrition, Department of Pediatrics, Faculty of Medicine, Hacettepe University, Ankara, Turkiye; 17 Division of Metabolic Diseases and Nutrition, Department of Pediatrics, Faculty of Medicine, İstanbul University – Cerrahpasa, İstanbul, Turkiye; 18 Division of Metabolic Diseases and Nutrition, Department of Pediatrics, Ankara Bilkent City Hospital, Ankara, Turkiye; 19 Division of Metabolic Diseases and Nutrition, Department of Pediatrics, Bursa Yüksek İhtisas Training and Research Hospital, University of Health Sciences, Bursa, Turkiye; 20 Division of Metabolic Diseases and Nutrition, Department of Pediatrics, Bağcılar Training and Research Hospital, İstanbul, Turkiye; 21 Division of Metabolic Diseases and Nutrition, Department of Pediatrics, Faculty of Medicine, Ankara Yıldırım Beyazıt University, Ankara, Turkiye; 22 Division of Metabolic Diseases and Nutrition, Department of Pediatrics, Van Training and Research Hospital, University of Health Sciences, Van, Turkiye; 23 Division of Metabolic Diseases and Nutrition, Department of Pediatrics, Faculty of Medicine, Akdeniz University, Antalya, Turkiye; 24 Division of Metabolic Diseases and Nutrition, Department of Pediatrics, Behçet Uz Children Research and Training Hospital, University of Health Sciences, İzmir, Turkiye; 25 Division of Metabolic Diseases and Nutrition, Department of Pediatrics, Faculty of Medicine, Kocaeli University, Kocaeli, Turkiye; 26 Division of Metabolic Diseases and Nutrition, Department of Pediatrics, Ankara Etlik City Hospital, Ankara, Turkiye; 27 Division of Gastroenterology, Hepatology and Nutrition, Department of Pediatrics, Faculty of Medicine, Başkent University, Ankara, Turkiye

**Keywords:** Acid maltase deficiency, Delphi study, expert opinion, infantile-onset Pompe disease, Turkish consensus

## Abstract

**Background/aim:**

Pompe disease (acid maltase deficiency, glycogen storage disease type II, OMIM #606800) is an autosomal recessive disorder characterized by lysosomal acid-α-glucosidase deficiency. The infantile-onset type of the disease is mainly characterized by cardiomegaly, hypotonia, and a high mortality rate. This study aimed to create a national consensus about infantile-onset Pompe disease (IOPD) to raise awareness among clinicians and standardize diagnosis and treatment approaches in Türkiye.

**Materials and methods:**

The Gazi University Division of Metabolic Diseases and Nutrition developed this expert opinion consensus and expanded it to include metabolism specialists across Türkiye. A systematic literature review was performed, and the Delphi method was used to evaluate the results.

**Results:**

Seventeen conclusive questions were produced about clinical presentation, diagnosis, and treatment, and 14 reached a consensus. Consensus was reached that general hypotonia is one of the most important findings, and agreement was also achieved on the starting dose of treatment for presymptomatic patients. The contributors agreed that gene therapy is a good treatment option for IOPD in the future.

**Conclusion:**

The topics related to this consensus will help physicians in Türkiye and elsewhere with high incidence rates of IOPD, especially regarding diagnosis and treatment decisions.

## 1. Introduction

Pompe disease (acid maltase deficiency, glycogen storage disease type II, OMIM #606800) is an autosomal recessive disorder characterized by lysosomal acid-α-glucosidase (GAA) deficiency [1]. GAA hydrolyzes lysosomal glycogen, and its deficiency results in glycogen accumulation in the lysosome, causing clinical symptoms, organ failure, and, in some cases, death [2]. There is a wide phenotypic variation depending on the age of onset, clinical presentation, enzyme activity, and organ involvement. The disease is classified into two main types: infantile-onset (classic/nonclassic) and late-onset (childhood/juvenile/adult), according to the age of symptom onset [1–4]. The infantile-onset type is mainly characterized by cardiomegaly, weakness, and hypotonia, with high mortality. The late-onset type is a slowly progressive myopathy involving skeletal muscle without cardiomyopathy [2,5,6].

Pompe disease is a rare metabolic disease with an incidence of 1/40,000 [7]. Before 2006, there was no option other than supportive treatment when it was first recognized; however, the life expectancy of patients has been prolonged, and their quality of life has increased with enzyme replacement therapy since then. As a result, its recognition has gained more importance. Due to the high prevalence of consanguineous marriages in Türkiye, we have frequently observed autosomal recessive disorders like Pompe disease in our outpatient clinics.

This study aims to create a national consensus about infantile-onset Pompe disease (IOPD) to raise awareness among clinicians, improve diagnosis, and standardize treatment approaches in Türkiye.

## 2. Materials and methods

### 2.1. Study design

This expert opinion consensus was developed by the Gazi University Division of Inborn Errors of Metabolism; it was later expanded to include metabolism specialists all over Türkiye. The study was conducted between April 2021 and May 2022. When the research was conducted, 40 centers following IOPD patients were asked whether they wanted to participate in the study through an online questionnaire. Twenty-nine centers responded positively. After further identifying these participating centers, an online recommendation questionnaire was sent to them using Google Forms. In centers with more than one specialist, a questionnaire was sent to the most senior doctor. The individuals who contributed to this study have followed up more than 100 IOPD patients. The Delphi method was used to evaluate the results [8].

### 2.2. Literature review

We performed a systematic review of publications containing information about IOPD. Case reports, guidelines, metaanalyses, clinical trials, randomized controlled trials, reviews, systematic reviews, and multicenter studies published from the beginning of 2016 until April 2021 were selected using PubMed’s bibliographic database and subsequently reviewed. Language restrictions were initiated, and only studies published in English and studies involving human subjects were included. The literature search was expanded to include ‘Pompe Disease,’ ‘Acid Maltase Deficiency,’ and ‘Glycogen Storage Disease Type 2.’

After initiating these searches, 179 publications were obtained. Twenty publications were unrelated to the topic, and the abstracts of 24 publications were inaccessible. Forty-eight publications were related to late-onset Pompe disease (LOPD), and 87 were on IOPD. The abstracts of the latter group were reviewed by one author, and 45 were included in the study. Additionally, five publications were added to the first literature review group according to the recommendations of several authors linked to this study. After the first literature review, 50 publications were deemed suitable for the study.

In the second literature review, 26 out of 50 publications were selected, and three new publications were added. After an independent metabolic specialist evaluated the first and second literature reviews, one publication was included. Finally, 30 publications eligible for inclusion in the study were obtained. The questionnaire used in the Delphi method was created with these publications in mind.

### 2.3. Questionnaire development and evaluation of results

The relevant texts of the chosen publications were read, and recommendations about clinical presentation, diagnosis, treatment, and follow-up were noted. Consensus on follow-up was determined as the subject of the next study. In this consensus, the first questionnaire, consisting of clinical presentation, diagnosis, and treatment, was created and sent to the participating centers using Google Forms in February 2022. The first questionnaire (Appendix I) consisted of 17 questions, and the respondents were allowed to tick more than one answer. Consensus was reached on responses that received ≥70% among responses in the first round; these were not included in the second questionnaire. The answers that received 50%–70% reached a consensus limit; therefore, the respondents voted again in the second questionnaire, while those that received ≤50% were not included. In the second round, the participants were allowed to mark an option titled ‘My decision has not changed.’ The second questionnaire (Appendix II) was sent in April 2022. For the responses in this second round, consensus was reached on responses that received ≥70%.

The results were received and evaluated on Google Forms. Twenty-five centers responded in the first round and 15 centers in the second. At the end of the first and second rounds, a consensus was reached on answers receiving 70% or more, and the article was prepared based on this consensus. The consensus guideline was developed without external financial support from industries or outside authors. The authors of this study only contributed academically.

## 3. Results and discussion

### 3.1. Clinical presentation

Pompe disease, also known as glycogen storage disease type 2 or acid maltase deficiency, is a rare, progressive, and often fatal lysosomal storage disorder caused by GAA deficiency [1,2]. It is inherited autosomal recessively and has wide phenotypic variation [2]. Pompe disease is also classified as a glycogen storage disorder because of the glycogen accumulation in lysosomes caused by GAA deficiency [2,9,10]. The accumulation may lead to clinical effects, and the severity of the disease is associated with residual enzyme activity [2].

Question 1: Which of the following sign/symptom(s) would you consider to be the most prominent criterion/criteria of IOPD?

Statement 1: General hypotonia, poor sucking, and frequent lower respiratory tract infections are the most prominent criterion/criteria of IOPD.

There are two types of Pompe disease: a rapidly progressive infantile-onset and a more slowly progressive late-onset type [2]. Cardiomegaly, hypotonia, weakness, motor delay, hepatomegaly, respiratory problems, and feeding problems are the initial symptoms of patients with IOPD [5,6,11]. Patients whose symptoms occur within the first year of life, who mostly expire due to cardiorespiratory failure, are classified as having ‘classic’ IOPD. Patients with milder symptoms occurring between the ages of 12–18 months are referred to as having ‘atypical’ IOPD [2,5,6,9]. However, it is important to remember that mild variant cases may appear before individuals are 12 months old [2]. It should also be noted that the symptoms mentioned in Statement 1 are highly suggestive of IOPD, but these symptoms are nonspecific and can also be seen in other muscle diseases. (See Appendix I for additional symptoms).

### 3.2. Diagnosis

Due to the rarity of the disease and the different severities of its symptoms, the first step is to correctly determine and diagnose the disease. In suspicious cases, the diagnosis should be confirmed by laboratory and molecular genetic analysis. In patients presenting with typical clinical symptoms, diagnosis is followed by demonstrating GAA deficiency and mutation analysis in the GAA gene [7,12]. In patients who apply to outpatient clinics or are hospitalized, in addition to routine laboratory tests such as complete blood count and biochemical analysis, creatine kinase (CK) analysis should also be performed if IOPD is suspected. Except for some LOPD patients, both infantile and LOPD patients have elevated CK. CK elevation is sensitive but nonspecific and should be considered with the symptoms and clinic presentation [2]. Aspartate aminotransferase (AST), alanine aminotransferase, (ALT) and lactate dehydrogenase may also be elevated and may suggest that these are being released from the muscles. Although transaminase elevation is sensitive but not specific, Pompe disease should be considered when persistent elevations occur [13].

Imaging methods are also used to support the diagnosis of Pompe disease, along with laboratory analysis. Chest X-ray, electrocardiogram (ECG), and echocardiogram are useful techniques for diagnostic algorithms [2]. Cardiomegaly can be seen in a chest X-ray [2]. In Pompe disease, ECG findings are visible due to the accumulation of glycogen in the cells of the cardiac conduction system [14]. Short PR intervals, a large QRS complex, large LV voltages, and increased QT dispersion can be observed on an ECG [2,15]. In a prior study, the authors demonstrated ventricular septum bulging and a different topography of the AV conduction system compared to normal anatomy. This may explain the ECG findings in Pompe disease, possibly caused by enlarged cells due to glycogen accumulation [14].

Hypertrophic cardiomegaly on echocardiography is typical for infantile-type Pompe disease. In the early stages, left ventricular outflow tract obstruction may accompany hypertrophic cardiomyopathy. Later, cardiac functions worsen, and dilated cardiomyopathy may develop [2].

Question 2: Which of the following routine laboratory analyses would you consider to be the primary criterion/criteria for determining IOPD?

Statement 2: Elevation in CK, combined elevation in CK, AST, ALT, the presence of cardiomegaly on telecardiography, the presence of a short PR interval OR high QRS wave on ECG, and the presence of right or left or biventricular hypertrophy on echocardiography would be considered primary criteria for determining IOPD.

In patients with suspected Pompe disease based on clinical and laboratory findings, GAA enzyme analysis is performed to confirm the diagnosis [2]. GAA activity can be measured in tissues such as cultured fibroblasts, muscle biopsy, mononuclear and lymphoid cells, and dried blood spots (DBS) [2]. Traditionally used muscle or fibroblast biopsies and lymphocytes do not exhibit maltase-glucoamylase activity, so it has been used for many years to measure GAA activity [16]. However, with the recent use of acarbose, which inhibits this enzyme [16–18], the measurement of GAA activity from DBS has become more widely used. Since the optimum activity of GAA occurred in acidic pH (3.7–4.5), its activity is measured in acidic pH with and without acarbose and compared with neutral glucosidase activity at a pH of 7.0 using glycogen and maltose or 4-methylumbelliferyl-α-D-glucoside (4-MUG), which is the maltose fluorescent synthetic analog as substrate [2,16]. DBS methods are noninvasive and rapid, and first-tier tests for Pompe disease diagnosis are advantageous as they require much less blood, stabilize lysosomal enzymes, and are easy to transport [2,16]. Although GAA enzyme analysis is usually <1% in IOPD, clinical and molecular genetic analysis is required to confirm the diagnosis [2].

Question 3: Which option(s) would you prefer regarding GAA enzyme assay to start treatment for infantile-type Pompe disease? Answer assuming you have access to all diagnostic tests for Pompe disease.

Statement 3: Acid alpha-glucosidase analysis in dry blood samples should be studied first, and positive results should be confirmed by intraleukocytic acid alpha-glucosidase analysis. If the patient’s condition is urgent and compatible with IOPD, the result of enzyme analysis from a DBS may be sufficient to start treatment.

GAA mutation analysis is used to confirm the diagnosis of Pompe disease [2]. The GAA gene has over 500 identified mutations and variants [2,19]. Single-base pair deletion [20], exon 18 deletion [20,21], and specific mutations due to founder effects, like 2741AG->CAGG insertion, previously seen in Turkish cases, can be determined [2].

Question 4: Which of the following (Appendix I) would you prefer for molecular analysis study methods in infantile-type Pompe disease?

Statement 4: GAA gene exon and exon-intron junctions sequence analysis should be performed in patients with clinically suspected infantile-type Pompe disease. If no mutation is detected in the sequence analysis of the GAA gene or if a single mutation is detected, the presence of large deletion-duplication should be investigated.

Question 5: Which of the following (Appendix I) would you agree with for the combined use of enzyme and molecular analyses to initiate clinical diagnosis and treatment?

Statement 5: Start with enzyme analysis, then confirm the positive results using molecular analysis.

The diagnosis of patients with Pompe disease, in addition to increased levels of CK, AST, and ALT, the value of glucotetrasaccharide (Glc4) (Glcα1-6Glcα1-4Glcα1-4Glc), a specific Glc4 excreted in urine, is steadily increasing [22]. Urinary Glc4 excretion can be elevated not only in Pompe disease but also in other glycogen storage diseases like type III and VI [23,24], Duchenne muscular dystrophy [23,25], and even during pregnancy [26]. Therefore, similar to CK, Glc4 elevation is sensitive but not specific [2]. However, Glc4 is a promising biomarker for Pompe disease [27,28], so it can be used as a noninvasive laboratory test before or in conjunction with enzymatic assay [27].

Question 6: Which biomarkers would you use to diagnose the disease in patients with clinically considered infantile Pompe disease? Answer assuming you have access to all diagnostic tests for Pompe disease.

Statement 6: Creatine kinase and Glc4 are used as biomarkers to diagnose patients with clinically considered infantile Pompe disease.

In a prior study, the change in urinary Glc4 excretion correlated with the clinical response to enzyme replacement therapy (ERT) [28]. This supports a previous hypothesis that glycogen accumulation in tissues and urinary Glc4 excretion correlate. The use of CK in evaluating treatment efficacy is limited due to its fluctuation [28] and elevation in other conditions, such as muscle diseases, cardiac diseases, and brain damage, and during surgery, injection, or while using certain drugs [29]. Consequently, urinary Glc4 excretion can be a potential biomarker in evaluating ERT efficacy [28]. In addition, since ERTs are biological agents, patients may develop antibodies against these drugs during follow-up, which can also determine the effectiveness of the treatment [2].

Question 7: Which biomarkers do you use in the follow-up treatment in patients with clinically considered infantile Pompe disease? Answer assuming you have access to all diagnostic/follow-up tests for Pompe disease.

Statement 7: Glc4 and antibodies to the drug are used as biomarkers in the follow-up treatment in patients with clinically considered infantile Pompe disease.

When evaluating treatment effectiveness, drug dosage and patient compliance are also factors that should be considered.

### 3.3. Treatment

Pompe disease is progressive and often fatal. Unfortunately, before ERT, a milestone in treating the disease, there was no treatment option other than symptomatic and supportive treatments [2]. ERT improves skeletal and cardiac muscle function and decreases cardiomegaly [2]. Intravenous recombinant human alglucosidase alfa (rh-GAA) is used as ERT and reduces glycogen storage by replacing the defective GAA responsible for the degradation of the alpha-1,4 and alpha-1,6 linkages [30].

Many factors affect ERT response: age at onset of treatment, disease severity at initiation of treatment, immune response, crossreactive immunological material (CRIM) status, and genotype [2]. Immune responses, one of the most important factors affecting ERT, significantly change the efficacy and safety of treatment [31,32]. In some patients, residual endogenous enzyme amounts exist, especially according to the mutation type, and this residual enzyme is termed as CRIM [31]. Patients with a residual enzyme with or without function are CRIM-positive, and those without it are CRIM-negative. CRIM status can be identified using a western blot combined with a pool of polyclonal or monoclonal antibodies raised against GAA [31,33]. The presence of this endogenous enzyme increases the tolerance of patients’ immune systems to ERT and affects the propensity to produce antidrug antibodies [31]. ERT causes a significant immune response and antibody development in CRIM-negative patients as it is perceived as foreign by the immune system [31]. This leads to an inadequate response to ERT [33,34]. In a study following 32 patients for 52 weeks, eleven of whom were CRIM-negative, the CRIM-positive patients showed better improvement cardiac and motor functions and overall survival was reduced; however, poor clinical outcomes were more pronounced among CRIM-negative patients. That study also determined that the development time of IgG antibodies against rh-GAA was faster, and the serotiters were higher in CRIM-negative patients [33]. All of these conditions are taken into consideration, and determining the CRIM status of patients becomes more important in terms of treatment effectiveness and course of the disease. There may be situations where the patient’s clinical findings and cardiac involvement are severe, requiring rapid initiation of treatment. In these cases, websites such as the Pompe Disease GAA Variant Database can also be used to determine the patient’s CRIM status.

Question 8: Which criterion/criteria would you consider regarding the determination of CRIM status before treatment in a patient diagnosed with infantile-type Pompe disease?

Statement 8: In an emergency case, starting the treatment without waiting for the CRIM result and then starting immunomodulation according to CRIM status.

The recommended treatment dose for rh-GAA, used since 2006, is 20 mg/kg/2 weeks [30]. In a study in which 18 IOPD patients diagnosed at 6 months of age were followed for 52 weeks, half received rh-GAA 20 mg/kg/2 weeks and the other half 40 mg/kg/2 weeks. All patients lived up to 18 months, and reduced risk of death, ventilation needs, and improvement in cardiomyopathy were observed. In addition, the enzyme has been shown to be safe and effective [35]. In the continuation of that study, 16 of the 18 patients continued the extended study. Half of the patients received ERT at 20 mg/kg/2 weeks, and the other half received 40 mg/kg/2 weeks up to 3 years. No difference in efficacy and safety was found between the 20 and 40 mg/kg/2 weeks doses [36].

In a cohort study with the European Pompe Consortium, data was collected from 124 classical IOPD patients from four countries with hypertrophic cardiomyopathy diagnosed before 12 months of age, and the difference between treatment doses was examined. In that study, 20 mg/kg every other week was used as the standard dose, 20 mg/kg per week or 40 mg/kg every other week as an intermediate dose, and 40 mg/kg per week as a high dose were examined. It was found that patients treated with the high dose of ERT (40 mg/kg per week) showed significant improvement in survival compared to those treated with the standard dose [37]. In another study, the initial treatment of IOPD patients was determined to be most effective at 40 mg/kg per week [38].

In our study, the consensus was 20 mg/kg/2 weeks for the starting dose of ERT in presymptomatic patients. There was no consensus on the starting dose of ERT for symptomatic patients. Fourteen study participants (n = 14/25; 56%) preferred 40 mg/kg/2 weeks as the starting dose for symptomatic patients, while nine (n = 9/25; 36%) chose 20 mg/kg/per week. Approximately 32% of the participants (n = 8/25) said they preferred to decide on a higher dose and dose frequency based on the patient’s situation. Most participants agreed on starting with an intermediate dose; therefore, it should be considered that the participants could choose from more than one option in the questionnaire.

Patients diagnosed through family history or neonatal screening programs are generally presymptomatic. Unfortunately, Pompe disease is not screened for within the scope of the neonatal screening program in Türkiye; therefore, most presymptomatic patients are diagnosed through family history or history of sibling death. In this Delphi study, the starting dose of treatment for presymptomatic patients was asked about and examined, but the time of initiation was not. Considering that IOPD is a progressive and life-threatening disease, it would be most beneficial for patients to start treatment as soon as possible.

Question 9: Which of the following options would you prefer regarding the starting dose of ERT (algucosidase alfa) in presymptomatic patients?

Statement 9: In presymptomatic patients, the starting dose of ERT (alglucosidase alfa) is 20 mg/kg/2 weeks.

Question 10: Which of the following options would you prefer regarding the starting dose of ERT (algucosidase alfa) in symptomatic (especially in terms of heart and respiratory system) patients?

Statement 10: Consensus could not be reached.

Pompe disease is characterized by multisystem effects, and patients should be evaluated in a multidisciplinary way during diagnosis and treatment. As mentioned before, patients may have cardiovascular, pulmonary, musculoskeletal, neurological, and gastrointestinal systems involvement with symptoms and signs related to these. In response to treatment, cardiac muscle responds better to ERT, unlike skeletal muscle response. Skeletal muscle response is mostly variable and positive if treatment is initiated before significant damage develops [2]. In a study in which patients were followed for long periods, the enzyme activity on survival, cardiomyopathy, ventilator status, and motor functions was evaluated [36]. These parameters are valuable in evaluating enzyme activity.

Question 11: Which system(s) do you primarily consider in infantile-type Pompe disease in terms of treatment goals?

Statement 11: In terms of treatment goals, the cardiovascular system and skeletal muscle are primarily considered.

Similarly, in our consensus, the primary system of treatment goals was determined as the cardiovascular system and skeletal muscle; however, consensus could not be reached on the secondary system to be evaluated in the first questionnaire. Among the responses to the first questionnaire, 64% of the participants (n = 16/25) pointed out that the involvement of sensory organs, such as hearing, should be considered secondary in terms of treatment effectiveness, while 60% (n = 15/25) stated that gastrointestinal system involvement is important. In the second questionnaire, a consensus was reached on sensory organs, such as hearing, as a system of secondary importance in terms of treatment goals. With the effectiveness of ERT and the prolongation of patient survival, the presence of non-life-threatening symptoms and the life quality of the patients become more important in evaluation and follow-up regarding treatment efficacy.

Question 12: Which system(s) do you consider as secondary in infantile-type Pompe disease in terms of treatment goals?

Statement 12: In terms of treatment goals, sensory organs, such as hearing, are considered secondary.

In a study involving the European Pompe Consortium, clinical deterioration was practiced in most patients followed up with the standard ERT dose of 20 mg/kg/2 weeks worldwide [37]. In the same study, 28 of 116 patients who received ERT had an increase in the treatment dose during follow-up. Deterioration of the patient’s situation, inadequate response, respiratory insufficiency, evidence in the literature, and the clinician’s personal experience are among the reasons to increase the dose [37]. In a different study in which 11 Pompe patients were followed, seven of whom had infantile-type Pompe disease, it was observed that a high dose of ERT is an option if there is a clinical plateau, inadequate response to treatment, or clinical worsening under treatment. The authors also emphasized that a high dose of ERT is required in cases where the muscle response is stable under treatment [38].

Question 13: In which system/systems do you increase the dose/frequency of treatment when the response you expect for ERT does not occur?

Statement 13: If there is no expected response to ERT in the heart and skeletal muscle, the dose/frequency of treatment should be increased.

In the literature, consensus studies can be found that promote the stopping criteria for ERT in LOPD [39,40], but there is no consensus for IOPD. Consistent with the literature, a consensus could not be reached for the stopping criteria for ERT in our study; 52% (n = 13/25) of the participants stated that they would stop the treatment in the presence of irreversible central nervous system damage, while 28% (n = 7/25) stated that they would never stop. The presence of conditions such as treatment response, family expectation, clinical experience, clinical status of the patients, and irreversible damage may require the termination of treatment to be considered.

Question 14: What is/are your criterion/criteria for stopping ERT?

Statement 14: Consensus could not be reached.

As mentioned before, the immune response to treatment is a crucial factor affecting the response to ERT [2]. There are two types of immunological reactions against ERT: infusion-associated reactions and IgG-mediated specific antibody development against the drug. Hypersensitivity reactions can be allergic (IgE or non-IgE mediated) or nonallergic and present with various symptoms, including cardiac, pulmonary, gastrointestinal, or skin manifestations [41].

One study suggested that treatment be given more slowly in a high-risk situation, such as an acute illness during infusion. In the same study, although routine premedication was not recommended, antihistamines, glucocorticoids, and tranexamic acid were recommended in cases where premedication was needed. If infusion reactions persist despite premedication, desensitization protocols are recommended [41].

In a study in which 14 patients were followed up, it was observed that four out of 13 patients who received ERT developed anaphylaxis. While a desensitization protocol was applied to all four patients, only one was premedicated. Methylprednisolone, diphenhydramine, and ranitidine were administered as premedication. Diphenhydramine and ranitidine were given together with methylprednisolone 1 h before infusion, and methylprednisolone was administered 13 h and 7 h before infusion [42]. In another study, in which three out of nine Pompe patients developed hypersensitivity reactions during ERT, it was stated that different combinations of paracetamol, hydroxyzine, and methylprednisolone were used as premedication. Montelukast was used in addition to the treatment in two patients who developed anaphylaxis. In the same study, the authors observed that the desensitization protocol was applied in all patients [43].

Question 15: What is your general protocol for infusion reactions?

Statement 15: Consensus could not be reached.

In our study, 56% (n = 14/25) of the participants chose ‘Antipyretic and/or antihistamine and/or steroids shortly before infusion,’ and 52% (n = 13/25) chose ‘I can use all of them depending on the frequency and severity’ for infusion reactions. There was also no consensus about this question in the second session. Considering the patient’s current situation and the recommendations of the pediatric allergy and immunology department, a patient-specific treatment can be developed.

Question 16: What is/are your preferences when anaphylaxis develops against ERT?

Statement 16: Desensitization is performed when anaphylaxis develops against ERT.

Consistent with the literature [42,43], a consensus was reached in our study on applying a desensitization protocol in case of anaphylaxis.

ERT is a revolutionary method for treating and following up on Pompe disease. This treatment prolonged patients’ survival, and improved cardiomyopathy and motor skill gains were observed [2,44]. However, Pompe disease cannot be completely cured with rh-GAA; therefore, studies have focused on developing new treatment approaches in recent years.

Mannose-6-phosphate receptors (CI-MPR) play an important role in the uptake of rh-GAA in muscle fibers, [44] so avalglucosidase alpha has been evaluated as a new treatment option with enhanced CI-MPR targeting [45,46]. In a prior study, the efficacy and safety of avalglucosidase alpha therapy were evaluated in 22 IOPD patients younger than 18 years of age who showed a clinical decline (cohorts 1 and 2) and suboptimal response (cohort 3) under alglucosidase alpha therapy. In that study, six patients were included in the first cohort and five in the second. Avalglucosidase alpha therapy was administered at 20 mg/kg/2 weeks and 40 mg/kg/2 weeks, respectively. In the third cohort, five patients received avalglucosidase alpha at 40mg/kg/2 weeks, and six patients continued with their current alglucosidase alpha doses. The patients were followed for 25 weeks, and improvement in motor functions was found to be better for those who received 40 mg/kg/2 weeks of avalglucosidase alpha than those receiving 20 mg/kg/2 weeks of avalglucosidase alpha or up to 40 mg/kg/week of alglucosidase alpha. In that study, avalglucosidase alpha was shown to have a positive clinical effect in IOPD [46].

A novel recombinant human rh-GAA-termed cipaglucosidase alpha combined with miglustat is undergoing clinical trials in IOPD patients, but no studies have been published yet [44]. Albuterol, a beta-agonist that enhances the expression of CI-MPR in the muscle cell’s membrane, has been used with alglucosidase alfa [44]. In a study with IOPD patients, albuterol was found to be safe but had limited benefits [47]. Treatment studies on pharmacological chaperones that can correct the defects in the affected protein are also ongoing [2,44].

Although ERT is a milestone in treating Pompe disease, as the patient’s survival rate increases, the treatment expectations also change. Studies on gene therapies will gain more importance in the future because ERT requires hospital admission, its tissue uptake is poor, it cannot cross the blood-brain barrier, it can cause variable immune responses, and it is a life-long therapy [48]. Lentiviral vectors for ex vivo hematopoietic stem cell gene therapy and adeno-associated viral vectors for in vivo gene therapy are newly developing, and it is expected that a cure will be found soon for Pompe disease [48].

Question 17: Which of the following treatment options, in your opinion, can be expected to have higher efficacy and safety potential than alglucosidase alfa in the near future?

Statement 17: Gene therapy will be a good treatment option in the near future.

## 4. Conclusion

This consensus study was developed as a result of a national collaboration of metabolism specialists focusing on IOPD in Türkiye. It aims to facilitate and standardize the diagnosis, treatment, and management of patients affected by IOPD throughout the country, regardless of their access. The study also aims to make it easier to determine or recognize IOPD and refer patients to a metabolism specialist, even if they have applied to a different medical department. In this study, symptoms occurring because of sleep problems and central nervous system, gastrointestinal system, and sensory organ involvement that patients may encounter during follow-up, as well as the management of these conditions, are not mentioned. Although the lack of this information can be considered a limitation, it is potentially the subject of a future study evaluating the long-term follow-up and management details of patients with IOPD. The Consensus Development Group aims to revise this study every 5 years to evaluate and share new research and therapies.

## Appendix I

### CLINICAL PRESENTATION

1. Which of the following sign/symptom(s) would you consider to be the ***most prominent*** criterion/criteria of IOPD? (You can tick more than one option)

- Proximal muscle weakness
- Peripheral hypotonia
- Axial hypotonia
- General hypotonia
- Poor sucking
- Frequent upperrespiratory tract infections
- Frequent lower respiratory tract infections
- Swallowing dysfunction
- Gastroesophageal reflux
- Swallowing dysfunction, feeding difficulties with gastroesophageal reflux
- Tongue fasciculation
- Macroglossia
- Hearing loss
- Hepatomegaly
- Decrease/loss of deep tendon reflexes
- Consanguinity

### DIAGNOSIS

2. Which of the following ***routine*** laboratory analyses would you consider to be the ***primary criterion/criteria*** for determining IOPD? (You can tick more than one option)

- Elevation of Creatine Kinase (CK)
- Elevation of Aspartate Aminotransferase (AST)
- Elevation of Alanine Aminotransferase (ALT)
- Combined elevation of Creatine Kinase, AST, ALT
- Presence of cardiomegaly on telecardiography
- Presence of short PR interval OR high QRS wave on electrocardiogram
- Presence of short PR interval AND high QRS wave on electrocardiogram
- Presence of left ventricular hypertrophy on echocardiography
- Presence of right or left or biventricular hypertrophy on echocardiography

3. Which option(s) would you prefer regarding acid alpha-glucosidase enzyme assay to start treatment for infantile type Pompe disease? Answer assuming you have access to all diagnostic tests for Pompe disease. (You can tick more than one option)

- Acid alpha-glucosidase enzyme analysis, which is studied by fluorimetric method in dry blood sample, should be preferred as the first analysis and is sufficient for a definitive diagnosis.
- Intraleukocyte acid alpha-glucosidase enzyme analysis should be preferred as the first analysis and is sufficient for a definitive diagnosis.
- Intra-tissue (fibroblast sample) acid alpha-glucosidase enzyme analysis should be preferred as the first analysis and is sufficient for definitive diagnosis.
- Acid alpha-glucosidase analysis in dry blood sample should be studied first, positive results should be confirmed by intraleukocyte acid alpha-glucosidase analysis.
- If the patient’s condition is urgent and compatible with infantile type Pompe disease, the result of enzyme analysis from a dry blood sample may be sufficient to start treatment.
- If the patient’s condition is urgent and compatible with infantile type Pompe disease, treatment can be started without enzyme analysis.

4. Which of the following would you prefer for molecular analysis study methods in infantile-type Pompe disease? (You can tick more than one option)

- GAA gene exon and exon-intron junctions sequence analysis should be performed in patients with clinically suspected infantile type Pompe disease.
- If no mutation is detected in the sequence analysis of the GAA gene or if a single mutation is detected, the presence of large deletion-duplication should be investigated.
- Direct new generation DNA sequence analysis should be performed to exclude other infantile diseases (eg SMA, Danon, etc.) whose clinical findings may overlap with infantile type Pompe disease and to start treatment as soon as possible.
- A direct whole exome analysis should be performed to exclude other infantile diseases (eg SMA, Danon, etc.) whose clinical findings may overlap with infantile type Pompe disease.
- Direct whole genome analysis should be performed to exclude other infantile diseases (eg SMA, Danon, etc.) whose clinical findings may overlap with infantile type Pompe disease.

5. Which of the following would you agree with for the combined use of enzyme and molecular analyses to initiate clinical diagnosis and treatment?

- Enzyme analysis is sufficient.
- Molecular analysis is sufficient.
- I start with enzyme analysis, I do molecular analysis to confirm the positive result.
- I start with molecular analysis, I do enzyme analysis to confirm the positive result.
- I start with molecular analysis, I do enzyme analysis when I encounter a change of unknown pathogenicity (VUS).

6. Which biomarkers would you use to diagnose the disease in patients with clinically considered infantile Pompe disease? Answer assuming you have access to all diagnostic tests for Pompe disease (You can tick more than one option).

- Complete blood count
- Creatine kinase
- AST and ALT
- Glucotetrasaccharide (Glc4)
- BNP (brain natriuretic peptide)

7. Which biomarkers do you use in the follow-up treatment in patients with clinically considered infantile Pompe disease? Answer assuming you have access to all diagnostic/follow-up tests for Pompe disease (You can tick more than one option).

- Complete blood count
- Creatine kinase
- AST and ALT
- Glucotetrasaccharide (Glc 4)
- BNP (brain natriuretic peptide)
- Antibodies to the drug

### TREATMENT

8. Which criterion/criteria would you consider regarding the determination of CRIM status before treatment in a patient diagnosed with infantile-type Pompe disease? (You can tick more than one option).

- Regardless of the clinical condition of the patient, I always check CRIM before treatment, if it is negative, I apply immunomodulation, if it is positive, I do not.
- In an emergency case, I start the treatment without waiting for the CRIM result, then I start immunomodulation according to the CRIM status.
- In an emergency case, I start the treatment without waiting for the CRIM result, I start immunomodulation according to the antibody levels against the drug.
- In an emergency case, I start treatment without waiting for a CRIM result, I start immunomodulation according to the clinical response to enzyme replacement therapy.
- I don’t check for CRIM at the beginning, I think about starting immunomodulation later according to the patient’s response to treatment and/or antibody levels to the drug.
- I start immunomodulation each patient regardless of their CRIM status.
- If there is an increase in antibodies to the drug and/or clinical unresponsiveness in a patient with CRIM-positive during treatment, I apply immunomodulation.
- I do not start immunomodulation in a patient for whom I started enzyme replacement therapy before the CRIM result came, if CRIM was negative, but I reached the treatment goals.

9. Which of the following options would you prefer regarding the starting dose of ERT (algucosidase alfa) in presymptomatic patients? (You can tick more than one option).

- 20 mg/kg/two weeks.
- 20 mg/kg/week
- 40 mg/kg/two weeks
- 40 mg/kg/week

10. Which of the following options would you prefer regarding the starting dose of ERT (algucosidase alfa) in symptomatic (especially in terms of heart and respiratory system) patients? (You can tick more than one option).

- 20 mg/kg/two weeks.
- 20 mg/kg/week
- 40 mg/kg/two weeks
- 40 mg/kg/week
- 60 mg/kg/two weeks
- 60 mg/kg/week
- I can follow up with higher dose and frequent application depending on the situation.

11. Which system(s) do you ***primarily*** consider in infantile-type Pompe disease in terms of treatment goals? (You can tick more than one option).

- Heart
- Lungs
- Skeletal muscle
- Gastrointestinal system
- Sense organs (hearing, etc.)
- Central and peripheral nervous system

12. Which system(s) do you consider as ***secondary*** in infantile-type Pompe disease in terms of treatment goals? (You can tick more than one option).

- Heart
- Lungs
- Skeletal muscle
- Gastrointestinal system
- Sense organs (hearing, etc.)
- Central and peripheral nervous system

13. In which system/systems do you increase the dose/frequency of treatment when the response you expect to for ERT does not occur? (You can tick more than one option).

- Heart
- Lungs
- Skeletal muscle
- Gastrointestinal system
- Sense organs (hearing, etc.)
- Central and peripheral nervous system

14. What is/are your criterion/criteria for stopping ERT? (You can tick more than one option).

- Irreversible deterioration in cardiac functions
- The need for mechanical respiratory support for a long time
- Irreversible central nervous system damage (due to anoxia etc.)
- Family want to discontinue enzyme replacement therapy
- Inadequate response despite applying the highest dose and frequency of enzyme replacement therapy you have determined.
- Severe allergic reaction
- Very high levels of antibodies to the drug and not responding to immunomodulation
- I never stop enzyme replacement therapy

15. What is your general protocol for infusion reactions?

- Antipyretic and/or anthistamine and/or steroid shortly before infusion
- Antipyretic and/or anthistamine and/or steroid shortly before and during the infusion
- Antipyretic and/or anthistamine and/or steroid shortly before, during and after the infusion
- Steroid the night before the infusion, antipyretic and/or anthistamine and/or steroid shortly before, during and after the infusion (Burton protocol)
- I can use all depending on the frequency and severity of infusion reactions

16. What is/are your preferences when anaphylaxis develops against ERT? (You can tick more than one option).

- I apply desensitization
- I administer omalizumab
- I take a break from enzyme replacement therapy for a while and then start again
- I terminate enzyme replacement therapy

17. Which of the following treatment options, in your opinion, can be expected to have higher efficacy and safety potential than alglucosidase alfa in the near future?

- Avalglucosidase
- AAV gene therapy
- Chaperone + Enzyme replacement therapy

## Appendix II

### CLINICAL PRESENTATION

1. Which of the following sign/symptom(s) would you consider to be the *most prominent* criterion/criteria of IOPD? (You can tick more than one option)

- MY DECISION HAS NOT CHANGED
- Poor sucking
- Frequent lower respiratory tract infections
- Macroglossia
- Decrease/loss of deep tendon reflexes

### DIAGNOSIS

2. Which of the following *routine* laboratory analyses would you consider to be the *primary criterion/criteria* for determining IOPD? (You can tick more than one option)

- MY DECISION HAS NOT CHANGED
- Combined elevaion of Creatine Kinase, AST, ALT
- Presence of short PR interval OR high QRS wave on electrocardiogram
- Presence of left ventricular hypertrophy on echocardiography

3. Which option(s) would you prefer regarding acid alpha-glucosidase enzyme assay to start treatment for an infantile type of Pompe disease? Answer assuming you have access to all diagnostic tests for Pompe disease (You can tick more than one option)

- MY DECISION HAS NOT CHANGED
- Acid alpha-glucosidase analysis in dry blood sample should be studied first, positive results should be confirmed by intraleukocyte acid alpha-glucosidase analysis.

4. Which biomarkers would you use to diagnose the disease in patients with clinically considered infantile Pompe disease? Answer assuming you have access to all diagnostic tests for Pompe disease (You can tick more than one option).

- MY DECISION HAS NOT CHANGED
- AST and ALT

5. Which biomarkers do you use in the follow-up treatment in patients with clinically considered infantile Pompe disease? Answer assuming you have access to all diagnostic/follow-up tests for Pompe disease (You can tick more than one option).

- MY DECISION HAS NOT CHANGED
- Creatine kinase
- Antibodies to the drug

### TREATMENT

6. Which of the following options would you prefer regarding the starting dose of ERT (algucosidase alfa) in symptomatic (especially in terms of heart and respiratory system) patients? (You can tick more than one option).

- MY DECISION HAS NOT CHANGED
- 40 mg/kg/two weeks

7. Which system(s) do you *primarily* consider in infantile-type Pompe disease in terms of treatment goals? (You can tick more than one option).

- MY DECISION HAS NOT CHANGED
- Lungs
- Skeletal muscle

8. Which system(s) do you consider as *secondary* in infantile-type Pompe disease in terms of treatment goals? (You can tick more than one option).

- MY DECISION HAS NOT CHANGED
- Gastrointestinal system
- Sense organs (hearing, etc.)

9. In which system/systems do you increase the dose/frequency of treatment when the response you expect to ERT does not occur? (You can tick more than one option).

- MY DECISION HAS NOT CHANGED
- Lungs

10. What is/are your criterion/criteria for stopping ERT? (You can tick more than one option).

- MY DECISION HAS NOT CHANGED
- Irreversible central nervous system damage (due to anoxia etc.)

11. What is your general protocol for infusion reactions?

- MY DECISION HAS NOT CHANGED
- Antipyretic and/or anthistamine and/or steroid shortly before infusion
- I can use all depending on the frequency and severity of infusion reactions
